# Glucocorticoids induce femoral head necrosis in rats through the ROS/JNK/c‐Jun pathway

**DOI:** 10.1002/2211-5463.13037

**Published:** 2020-11-30

**Authors:** Puji Peng, Zhigang Nie, Fei Sun, Hao Peng

**Affiliations:** ^1^ Department of Orthopedics Renmin Hospital of Wuhan University China

**Keywords:** apoptosis, autophagy, hormonal osteonecrosis of the femoral head, JNK/c‐Jun signaling pathway, ROS

## Abstract

Osteonecrosis of the femoral head (ONFH) is a common clinical disease with a high disability rate. Apoptosis of osteoblasts caused by high‐dose short‐term or low‐dose long‐term glucocorticoid (GC) administration is the biological basis of steroid‐induced avascular necrosis of the femoral head (SANFH). The pathogenesis of SANFH has not yet been fully elucidated, and there is currently a lack of effective clinical treatments. Here, we investigated the role of the reactive oxygen species (ROS)/JNK/c‐Jun signaling pathway in SANFH. Dexamethasone (Dex) was used to induce apoptosis in osteoblasts, and this resulted in a significant increase in levels of p‐JNK, p‐c‐Jun, Bax, caspase‐3, caspase‐9, cytochrome C, Beclin‐1, and LC3, and a decrease in levels of P62 and Bcl‐2. In addition, intracellular ROS levels were increased and mitochondrial membrane potential was decreased. Administration of 3‐MA, an autophagy inhibitor, attenuated Dex‐mediated changes in autophagy and apoptosis. A rat model of ONFH exhibited severe bone trabecular hollow bone pits along with a significant increase in femoral head cell apoptosis compared with the control group. Additionally, micro‐CT analysis showed that both bone tissue content and femoral head integrity were significantly reduced in the ONFH group. Furthermore, 3‐MA treatment decreased the effect of Dex on GC‐induced ONFH and osteoblast apoptosis in rats and could counteract microstructure destruction due to femoral head necrosis. In summary, our data suggest that GC can induce osteoblast apoptosis and autophagy through the ROS/JNK/c‐Jun signaling pathway, which contributes to ONFH.

## Graphical abstract

Apoptosis of osteoblasts caused by glucocorticoid (GC) is the biological basis of steroid‐induced avascular necrosis of the femoral head (SANFH). We found that GC can induce osteoblast apoptosis and autophagy through the ROS/JNK/c‐Jun signaling pathway, which contributes to SANFH. Therefore, the autophagy inhibitor 3‐MA might have potential for development into a treatment for SANFH.

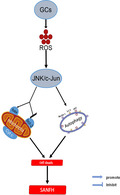

**Abbreviations**
ALP: alkaline phosphatase
BrdU: 5‐bromo‐2'‐deoxyuridine
Dex: Dexamethasone
GC: Glucocorticoid
HE: hematoxylin and eosin
ONFH: osteonecrosis of the femoral head
SANFH: steroid‐induced avascular necrosis of the femoral head

Osteonecrosis of the femoral head (ONFH) is a common clinical disease with a high disability rate [1]. Apoptosis of osteoblasts caused by high‐dose short‐term or low‐dose long‐term glucocorticoid (GC) administration is the biological basis of steroid‐induced avascular necrosis of the femoral head (SANFH) [2, 3]. The pathogenesis of SANFH has not yet been fully elucidated, and there is currently a lack of effective clinical treatments. Therefore, it is important to investigate the molecular mechanism underlying the occurrence and development of SANFH. Recent studies have found that GC‐induced osteoblast autophagy is related to ONFH [4]. However, the involvement of downstream signaling pathways in GC‐induced autophagy and apoptosis of osteoblasts needs to be further investigated. Based on this, we established *in vivo* and *in vitro* SANFH models to explore the role of GC in the process of SANFH induction and to provide new directions for potential clinical treatments for SANFH.

## Materials and methods

### Reagents and instruments

FBS was purchased from Sera Pro (Naila, Germany). Methylprednisolone was obtained from Pfizer (South San Francisco, CA, USA). Dexamethasone (Dex); LPS; SP600125; and primary antibodies against p‐JNK, c‐JUN, p‐c‐Jun, JNK, Bcl‐2, LC3, Caspase‐3, and Bax were purchased from Sigma (St. Louis, MO, USA). Penicillin–streptomycin solution was purchased from Servicebio (Wuhan, Hubei, China); MPS was purchased from Pfizer, N‐acetyl‐L‐cysteine, and CCK‐8 reagent were purchased from Beyotime Biotechnology (Beijing, China), and 3‐methyladenine was purchased from Selleck Chemicals (Houston, TX, USA). The CO_2_ incubator was purchased from Binder (Tuttlingen, Germany), and an ultra‐clean laboratory bench, surgical instruments, fluorescence microscope, and inverted microscope were purchased from Olympus (Tokyo, Japan).

### Animals

Male Sprague Dawley rats (*n* = 36) aged 8 weeks and weighing 220 ± 20 g were purchased from Charles River Laboratories (Beijing, China). The rats were randomly divided into three groups (*n* = 12/group): (a) a control group (CG), injected with 0.9% saline and fed for 1 week; (b) an ONFH group, injected intravenously with 2 mg·kg^−1^ LPS on the first 2 days and then injected intramuscularly with 20 mg·kg^−1^ MPS on days 3–7; and (c) a 3‐MA group (3‐MAG), injected intraperitoneally with 5 mg·kg^−1^ 3‐MA 2 h before MPS injection. The rats were housed under specific pathogen‐free conditions. Besides, they were in plastic cages which were maintained at 26 °C and 55% humidity. The mice had free access to food and water. Rats were housed in a photoperiod of 12 h of light and 12 h of darkness (from 20:00 h through 8:00 h). Rats were euthanized under anesthesia by intraperitoneal injection of 130 mg·kg^−1^ sodium pentobarbital on day 28, and femoral head tissues were collected. All attempts were made to minimize the suffering. The study was conducted according to the requirements of the Experimental Animal Management and Use Committee of Wuhan University and was approved by the Ethics Committee of Wuhan University (No. 20180920).

### Cell culture

Following anesthesia, we collected the skull, blood vessels, and connective tissue samples from each rat. Bone tissues were further cut into fragments and transferred to a culture flask, digested with trypsin (4 mL) for 5 min, and neutralized with serum‐containing medium. Cells were collected by centrifugation and were then incubated with 0.1% collagenase for 30 min. Next, primary osteoblasts were collected by centrifugation and cultured at 37 °C and 5% CO_2_ with Ham's F‐12 medium containing 10% FBS. All procedures were performed under sterile conditions using second to fifth generation osteoblasts. We divided the cell samples into the following five groups: control, Dex, Dex + NAC, Dex + SP600125, and Dex + 3‐MA.

### Cell viability

Osteoblasts were seeded into 96‐well plates at a density of 1 × 10^3^ cells/well. After 24 h of Dex treatment (absolute ethanol was used to dissolve Dex powder), we added 10 μL of CCK‐8 reagent to each well. Cell viability was determined by measuring the absorbance at 450 nm after 1 h of incubation at 37 °C.

### Cell proliferation

Osteoblasts were seeded into a 24‐well plate at a density of 3 × 10^3^ cells/well. To determine cell proliferation, 5‐bromo‐2'‐deoxyuridine (BrdU) solution was added dropwise to each well 3 h before Dex application.

### Apoptosis

The In Situ Cell Death Detection Kit (Roche, Basel, Switzerland) was used to detect apoptosis with TUNEL staining. Brownish‐yellow cells were identified as apoptotic cells.

### Alkaline phosphatase activity

Cells were seeded into a 12‐well plate at a density of 5 × 10^4^/well, exposed to different processing conditions, and then treated with 100 μL of lysis buffer after washing. The alkaline phosphatase (ALP) activity was measured using the ALP reagent.

### ROS detection

Cells were seeded into a 24‐well plate at a density of 3 × 10^3^/well and exposed to different processing conditions. After washing three times with PBS, the centrifuged cells were collected, and the cell pellet was suspended in a concentration of 10 μm DCFH‐DA (KeyGEN Biotech, Nanjing, China). Following 30 min of incubation at room temperature in the dark, flow cytometry was used to analyze the average fluorescence intensity.

### Mitochondrial membrane potential

We used a JC‐1 Assay Kit (Sigma) to evaluate ΔΨm. The cell pellet obtained by digestion and centrifugation was resuspended in 0.5 mL of culture medium. Next, 0.5 mL of JC‐1 staining working solution was added, following by mixing and incubation at 37 °C for 20 min. Thereafter, the samples were centrifuged at 300 ***g*** at 4 °C for 3–4 min. Then, the cells were resuspended in 1 mL of JC‐1 staining buffer and centrifuged. We repeated this process twice before the cells were resuspended with JC‐1 staining buffer, and the signals for the FL1 and FL2 channels (at an excitation wavelength of 488 nm) were measured using flow cytometry.

### Western blotting

We prepared the cell lysates to extract cell proteins from the control and experimental groups. After electrophoresis and transfer to polyvinylidene fluoride (PVDF) membranes, the PVDF membranes were blocked with 5% skimmed milk for 2 h and incubated with the primary antibody at 4 °C overnight. After washing three times with TBST, membranes were incubated with the secondary antibody at room temperature for 2 h. The membrane was washed three times with TBST to detect luminescence.

### Autophagy

Cells were seeded in 6‐well plates at a density of 3 × 10^4^ cells/well. After treatment, cells were washed twice with PBS. Then, 0.05 mm of MDC detection solution (Sigma) was added, and the cells were incubated at 37 °C for 10 min. Following incubation, the cells were washed twice with PBS. The average fluorescence intensity was observed and recorded under a fluorescence microscope.

### Hematoxylin and eosin (HE) staining

Femoral head tissue was fixed in formaldehyde solution (pH = 7.4) for 48 h and then decalcified in 10% EDTA solution for 4 weeks (NaOH was used to adjust the pH of EDTA solution to improve its solubility). Then, decalcified bone tissue was embedded in paraffin and cut into 5‐μm‐thick sections for HE staining and subsequent morphological evaluation.

### Micro‐CT scan analysis

Femoral heads were examined using micro‐CT with a thin layer scan (on the order of 20 µm) using a current of 160 μA and scanning voltage of 80 kV. We used the software provided with the system to quantitatively analyze the bone volume fraction (BV/TV), trabecular thickness (Tb.Th), trabecular space (Tb.Sp), trabecular number (Tb.N), etc.

### Extraction of total protein from bone tissues

Following anesthesia, we collected the skull, and then, the blood vessels and connective tissues were carefully removed as completely as possible. Bone tissues were transferred into a mortar and ground with liquid nitrogen. Powdered bone tissue was collected in a 1.5‐mL EP tube. Protein lysis solution was added to the EP tube (200 µL of lysis solution per 100 mg of bone tissue). Bone tissue was lysed on ice for 30 min. After centrifugation at 13 523 ***g*** at 4 °C for 15 min, the supernatant was collected as the total protein extract.

### Statistical analysis

All experiments were performed in triplicate, and the data were analyzed using graphpad (version 6.0, San Diego, CA, USA). Data are expressed as the mean ± SD, and comparisons between multiple groups were performed using ANOVA. *P* < 0.05 was considered statistically significant.

## Results

### Dexamethasone reduces the viability of rat osteoblasts and induces autophagy and apoptosis

**Fig. 1:**
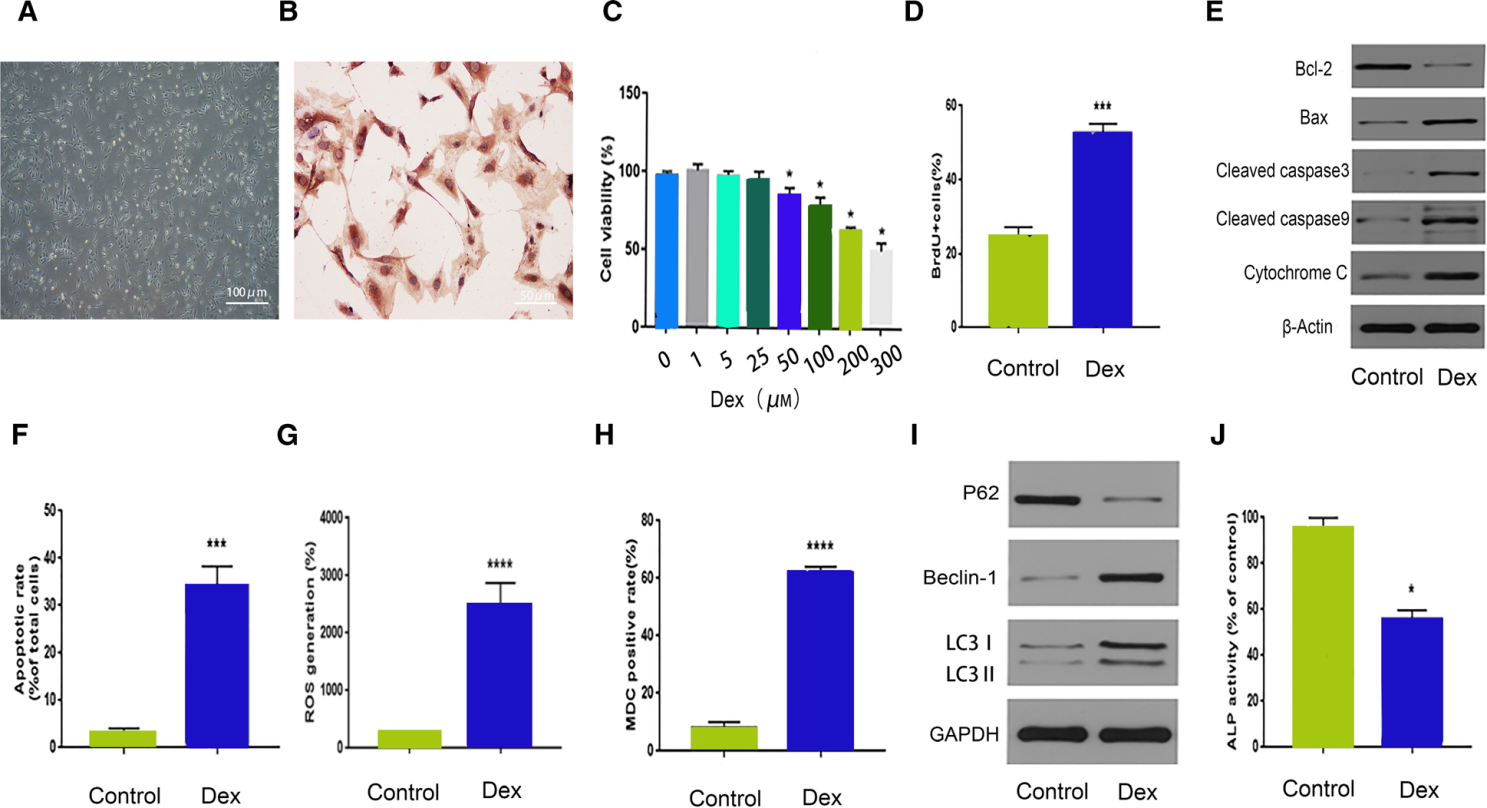
Dex reduces the viability of rat osteoblasts and induces apoptosis and autophagy. (A) Microscopic images of osteoblasts (Scale bar = 50 µm). (B) Immunohistochemical staining of type I collagen in osteoblasts (Scale bar = 100 µm). (C) Viability of osteoblasts after 24 h of Dex treatment (0, 1, 5, 25, 50, 100, 200, and 300 μm). (D) ALP activity of Dex‐treated osteoblasts. (E, F) Expression levels of apoptosis‐ and autophagy‐related proteins in Dex‐treated osteoblasts. (G) DCFH‐DA staining, (H) BrdU incorporation assay, (I) TUNEL staining, and (J) MDC staining for control and Dex‐treated osteoblasts. For (D–J), we used Dex at a concentration of 200 μm at 24 h. Compared with CG, *P < 0.05,***P < 0.001, ****P < 0.0001 (Student's *t*‐test). The quantitative statistics were presented as the mean ± SD (*n* = 3).

The rat primary osteoblasts showed fibroblast‐like morphology (Fig. 1A). Osteoblasts showed a high expression of type I collagen, which was dark brown in color (Fig. 1B). To investigate the effects of GC on the toxicity and viability of rat osteoblasts, we applied different concentrations of Dex (0, 1, 5, 25, 50, 100, 200, and 300 μm) to osteoblasts for 24 h. Culture medium was used as the solvent for Dex. Using the CCK‐8 assay, we found that Dex reduced osteoblast viability in a dose‐dependent manner, with an IC_50_ of 300 μm. Cell viability was significantly suppressed at a Dex concentration of 200 μm. Therefore, we selected a concentration of 200 μm Dex in subsequent experiments (Fig. 1C). BrdU proliferation experiments showed that Dex treatment significantly inhibited the proliferation ability of osteoblasts (Fig. 1D). In addition, the expression levels of apoptosis‐related proteins Bax, cleaved‐caspase‐3, cleaved‐caspase‐9, and cytochrome C were significantly upregulated, whereas the expression levels of Bcl‐2 were downregulated (Fig. 1E). TUNEL staining showed that the apoptosis rate of osteoblasts increased to 34.6% following Dex treatment (Fig. 1F). These results indicate that the cell apoptosis rate significantly increased following Dex treatment. In addition, DCFH‐DA staining showed that the average ROS fluorescence intensity of Dex‐treated osteoblasts increased compared to CG (*P* < 0.0001, Fig. 1G). To further investigate the effect of Dex on osteoblast autophagy, we measured the average fluorescence intensity of autophagic vesicles within 24 h of Dex treatment and found that the average fluorescence intensity significantly increased (*P* < 0.0001; Fig. 1H). We also detected the expression of a number of autophagy‐related proteins using western blot analysis. The protein levels of LC3B‐II, LC3B‐I, and Beclin‐1 were upregulated, whereas the protein level of P62 was downregulated (Fig. 1I), which is consistent with previous results. To evaluate the degree of osteoblast differentiation, we measured ALP activity. Dex significantly reduced ALP activity and inhibited osteoblast differentiation (Fig. 1J). Collectively, these results indicate that Dex can induce apoptosis and autophagy of rat osteoblasts.

### 3‐MA inhibits Dex‐induced osteoblast autophagy and reverses apoptosis

**Fig. 2:**
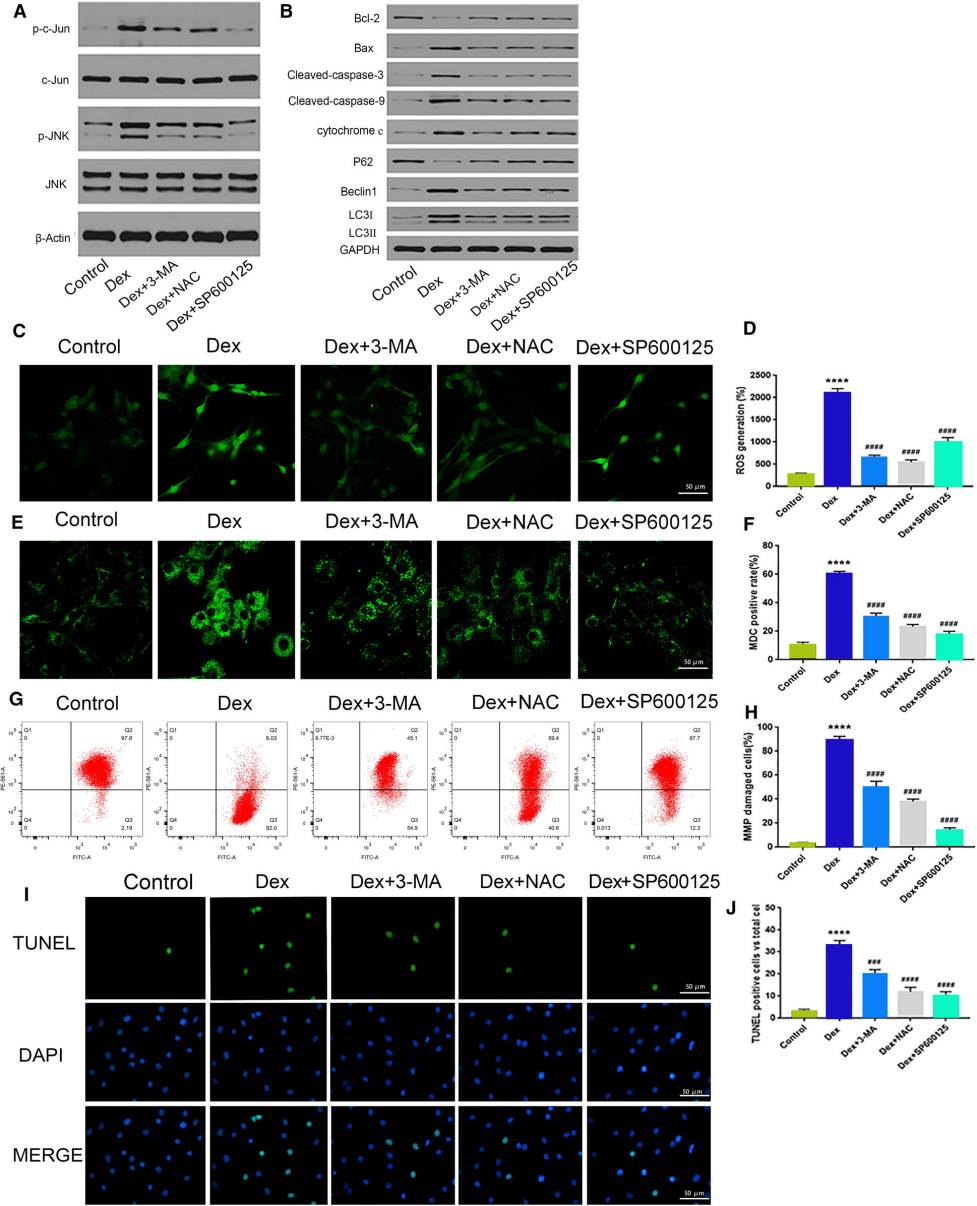
GC can induce osteoblast apoptosis and autophagy through the ROS/JNK/c‐Jun signaling pathway. Protein expression levels of (A) p‐c‐Jun, c‐Jun, p‐JNK, and JNK; (B) LC3B‐II, LC3B‐I, Beclin‐1, P62, Bax, caspase‐3, caspase‐9, bcl‐2, bax, and cytochrome C after Dex or Dex + NAC/3‐MA/SP600125 treatment in osteoblasts. (C, D) DCFH‐DA staining (Scale bar = 50 µm); (E, F) MDC staining (Scale bar = 50 µm); (G, H) V‐FITC/PI assay; and (I, J) TUNEL staining after Dex or Dex + NAC/3‐MA/SP600125 treatment in osteoblasts (Scale bar = 50 µm). For (A‐J), we used Dex at a concentration of 200 μm at 24 h. Compared with CG, ****P < 0.0001; ###*P* < 0.001, ####*P* < 0.0001 compared with the ONFH group (Student's *t*‐test). The quantitative statistics were presented as the mean ± SD (*n* = 3).

To examine the potential relationship between Dex‐induced osteoblast autophagy and apoptosis, we used the autophagy inhibitor 3‐MA in the next set of experiments. As shown in Fig. 2B, the expression levels of autophagy‐related proteins LC3B‐II, LC3B‐I, and Beclin‐1 decreased in Dex + 3‐MAG, whereas there was an increase in P62 protein expression in this group. The levels of ROS in osteoblasts after Dex treatment were measured using DCFH‐DA staining (Fig. 2C,D). The mean fluorescence intensities in osteoblasts were distinctly increased after Dex treatment, which suggested that Dex increased the ROS levels in osteoblast. NAC, SP600125, and 3‐MA were used as ROS scavengers in this study, which significantly decreased the levels of ROS in osteoblasts. The average fluorescence intensity of autophagic vesicles also decreased (Fig. 2E,F), along with a restoration of mitochondrial membrane potential (Fig. 2G,H) and a reduction in cell apoptosis (Fig. 2I,J). These results demonstrate that 3‐MA can significantly inhibit Dex‐induced osteoblast autophagy and reverse cell apoptosis.

### The ROS/JNK/c‐Jun signaling pathway mediates the effect of Dex on osteoblasts

To investigate whether the JNK/c‐Jun signaling pathway plays a role in the Dex‐induced effects in osteoblasts, we assessed protein expression levels of p‐JNK and p‐c‐Jun. Both proteins were significantly upregulated, which suggests that the JNK/c‐Jun signaling pathway is involved in Dex‐induced effects in rat osteoblasts. The involvement of the JNK/c‐Jun pathway was confirmed using the JNK inhibitor SP600125 to block the JNK/c‐Jun pathway. Changes in apoptosis‐ and autophagy‐related protein expressions were detected by western blot analysis. Following treatment with SP600125, the protein expressions of LC3B‐II, LC3B‐I, and Beclin‐1 decreased, whereas the expression of P62 increased (Fig. 2A,B). This change in protein expression was accompanied by a decrease in the average fluorescence intensity of autophagic vesicles (Fig. 2E,F). In addition, the mitochondrial membrane potential was restored (Fig. 2G,H), and the apoptosis rate decreased to 12.1% (Fig. 2I,J). These results confirm that the JNK/c‐Jun signaling pathway is involved in Dex‐induced cell autophagy and apoptosis in rat osteoblasts.

The role of ROS was further explored in Dex‐induced osteoblast autophagy and apoptosis. We measured various properties of osteoblasts following treatment with NAC, an inhibitor of ROS generation. The protein expression levels of p‐c‐Jun and p‐JNK were downregulated following NAC treatment. In addition, the expression of Bcl‐2 increased, whereas that of Bax and two apoptosis‐initiating proteins, caspase‐3 and caspase‐9, decreased (Fig. 2A,B). The mitochondrial membrane potential was also restored (Fig. 2I,J). These results suggest that ROS mediate the activation of the JNK/c‐Jun signaling pathway, promoting osteoblast autophagy and apoptosis. Thus, NAC can inhibit activation of the JNK/c‐Jun signaling pathway by reducing excessive ROS production, thereby reversing the cytotoxic effects of Dex.

### 3‐MA decreases GC‐induced ONFH and osteoblast apoptosis in rats

**Fig. 3:**
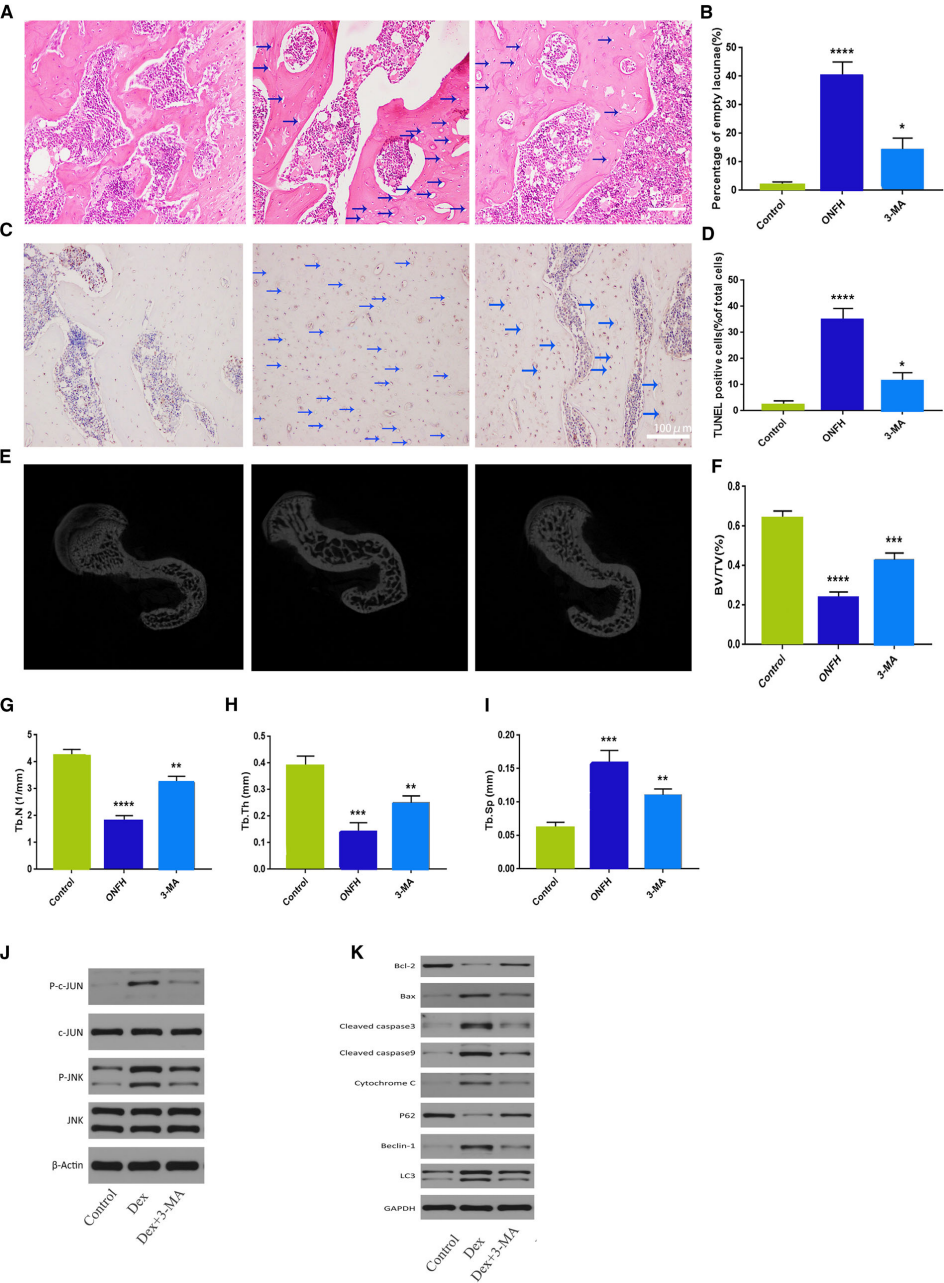
Evaluation of osteonecrosis and apoptosis in GC‐ and 3‐MA‐treated rats. (A) HE staining of femoral heads of rats in the CG, ONFH group, and 3‐MAG; blue arrows indicate empty lacunae (Scale bar = 50 µm). (B) Quantitative analysis of the empty lacunae rate in the three groups. (C) TUNEL staining of femoral heads of rats in the three groups; blue arrows indicate TUNEL‐positive cells (Scale bar = 100 µm). (D) Quantitative analysis of the proportion of TUNEL‐positive cells in the three groups. (E) Micro‐CT analysis of the CG, ONFH group, and 3‐MAG. Quantitative analysis of (F) BV/TV; (G) Tb.N; (H) Tb.Th; and (I) Tb.Sp in the three groups. Protein expression levels of (J) p‐c‐Jun, c‐Jun, p‐JNK, JNK; (K) LC3B‐II, LC3B‐I, Beclin‐1, P62, Bax, caspase‐3, caspase‐9, bcl‐2, bax, and cytochrome C after Dex or Dex + 3‐MA in osteoblasts. The magnification of HE staining and TUNEL staining is ×200 and ×400, respectively. A concentration of 200 μm of Dex was used for (A)‐(H) at 24 h. *P<0.1, **P < 0.01, ***P<0.001, ****P < 0.0001 compared with the CG (Student's *t‐*test). The quantitative statistics were presented as the mean ± SD (*n* = 3).

We conducted animal experiments to further verify the effects of 3‐MA on femoral head necrosis induced by GC in rats. No femoral head necrosis was observed in controls, whereas 8 of the 12 rats in the ONFH model group had bilateral femoral head necrosis and four had unilateral femoral head necrosis (necrosis rate, 20/24). Unilateral femoral head necrosis also occurred in 3 of the 12 rats in the 3‐MAG (necrosis rate of 3/24). HE staining of femoral head necrosis was characterized by trabecular bone fractures, disordered arrangement, and a large number of empty bone pits. The trabeculae in CG were complete and regularly arranged with osteocytes present in the bone dimples. Conversely, the bone trabeculae in the ONFH group were disordered, narrowed, or broken, with empty bone dimples apparent throughout. In 3‐MAG, the trabecular shape was better than that in the ONFH group as there was no obvious narrowing or fracture. Empty bone dimples were present in 3‐MAG but were fewer than those in the ONFH model group (Fig. 3A).The CG, ONFH group, and 3‐MAG had empty bone crater rates of 2.3%, 39.1%, and 19.2%, respectively (Fig. 3B).

We used TUNEL staining to detect apoptosis in rat femoral head cells (Fig. 3C). The rate of TUNEL‐positive cells was 30.2% in the ONFH model group and 9.7% in 3‐MAG. Thus, the proportion of apoptotic cells to normal cells was significantly lower in 3‐MAG than in the ONFH model group (Fig. 3D). These results further illustrate the inhibitory effect of 3‐MA on femoral head necrosis and osteoblast apoptosis.

### 3‐MA protects against the microstructure destruction in femoral head necrosis

Micro‐CT scanning is able to clearly show the microstructure of bone trabeculae, reflecting the structural integrity of tissues and changes in bone mass. Thus, it is by far the best method to evaluate the microstructure of bone tissue. Micro‐CT scanning and reconstruction analysis showed the trabeculae in CG were clearly visible, tightly connected, and the surface of the femoral head was smooth without collapse (Fig. 3E). In contrast, the ONFH group had sparse trabecular bones, with a greater distance between the trabecular bones, and a large number of trabecular bone microfractures. In 3‐MAG, the trabecular bones had smaller trabecular bone spacing compared with that in the ONFH group, and the arrangement was partially disordered with some trabecular bone microfractures (Fig. 3F–I). Overall, these results suggest that 3‐MA can significantly protect against microstructure destruction of the femoral head of rats, improving and normalizing the quantity and shape of the bone trabecula.

We extracted total protein from rat bone tissue and performed further *in vivo* studies to verify its mechanism. Compared with those in the CG, the expression levels of p‐JNK, p‐c‐Jun, caspase‐3, and Bax were upregulated after GC intervention, but the expression level of Bcl‐2 was downregulated. In addition, after GC intervention, the expression levels of autophagy‐related proteins LC3B‐II and Beclin‐1 were upregulated, that of P62 was downregulated, mitochondrial membrane potential decreased, and apoptosis and autophagy increased. 3‐MA treatment attenuated all these effects (Fig. 3J,K). These results suggest that activation of the JNK/c‐Jun signaling pathway plays an important role in GC‐induced osteoblast apoptosis and autophagy, which is consistent with our *in vitro* experimental results.

## Discussion

Although studies have shown that GC‐induced osteoblast autophagy and apoptosis are valuable in the pathogenesis of ONFH [4, 5, 6], the underlying mechanism of ONFH pathogenesis remained unknown. Unfortunately, ONFH can occur in 9–40% of patients with long‐term GC steroid use [7]. After the development of ONFH, the necrotic bone tissue is abnormally fragile, and given that the hip joint needs to bear weight, this will eventually cause the femoral head to collapse, thereby affecting the entire hip joint. The incidence of ONFH resulting in disability is very high. However, there are no effective nonsurgical treatments, even for the early stages of the disease. As the disease progresses, hip replacement surgery is required, and this is associated with a high level of pain and is a burden to patients and society [8]. Therefore, it is extremely important to study and better understand the pathogenesis of ONFH.

Cell autophagy, such as apoptosis and aging, is an extremely important biological phenotype considering its involvement in various processes of the body, including growth, development, metabolism, and differentiation [9, 10]. Cells are able to maintain normal metabolism through autophagy. However, autophagy can also induce or inhibit apoptosis. Indeed, a complex relationship exists between autophagy and apoptosis, and their coordination plays an important role in maintaining osteoblast homeostasis and survival [11, 12]. Edinger and Thompson demonstrated that GC‐induced osteoblast autophagy is closely related to ONFH [4]. GC can regulate expression of MC3T3‐E1 cell autophagy‐related proteins (Beclin‐1, LC3B‐II, and P62) and endoplasmic reticulum stress‐related proteins (ATF4 and CHOP). Thus, activation of autophagy and endoplasmic reticulum stress‐related pathways can ultimately lead to apoptosis. Our results showed that 3‐MA significantly inhibited cell autophagy and protected osteoblasts from Dex‐induced apoptosis.

Reactive oxygen species are mainly formed when mitochondria use free radicals in redox reactions. The intracellular antioxidant system and ROS play important regulatory roles in the differentiation and apoptosis of osteoblasts. Conversely, under pathological conditions, excess ROS can cause oxidative damage through mitogen‐activated protein kinase (MAPK) and P53 signaling pathways [13, 14]. De Meyer *et al*. [2] found that the increase in ROS caused by Dex plays a major role in activating cell autophagy and participates in osteoblast apoptosis. Furthermore, the proportion of autophagy and apoptosis was significantly reduced when NAC was used to eliminate excess ROS. These results confirm that excess ROS levels mediate the regulation of Dex‐induced autophagy and apoptosis in osteoblasts.

JNK is an important member of the MAPK family and has been shown to participate in oxidative stress, apoptosis, proliferation, and autophagy [15, 16]. Various stress factors (e.g., oxidative stress, mechanical stimulation, and starvation) lead to JNK activation, which in turn leads to c‐Jun phosphorylation, further promoting the release and expression of caspase‐3 and resulting in apoptosis [17]. JNK is involved in osteoblast development and plays an important role in bone formation [18, 19]. Zhang *et al*. [20] demonstrated that activation of the ROS/JNK signaling pathway in human osteosarcoma cells can induce apoptosis and autophagy. In the present study, we found that Dex activates the JNK/c‐Jun pathway by upregulating p‐JNK and p‐c‐Jun protein expression in osteoblasts. This activated JNK/c‐Jun pathway promotes Bax expression, which then moves to the mitochondria and combines with Bcl‐2 to form a dimer that promotes apoptosis. As a result, this increases the permeability of the mitochondrial membrane for cytochrome C, reduces mitochondrial membrane potential, and ultimately activates the mitochondrial apoptotic pathway.

We found that GC caused ROS production and expression of p‐JNK and p‐c‐Jun in osteoblasts and activated the JNK/c‐Jun signaling pathway, thereby inducing autophagy and apoptosis. The mechanism may involve the ROS/JNK/c‐Jun pathway. ROS inhibitors were used to further verify the upstream pathway of autophagy. After intervention with ROS inhibitors, we found that NAC can reduce ROS generation, inhibit the activation of the JNK/c‐Jun signaling pathway, and reverse apoptosis and autophagy in osteoblasts induced by GC. We also found that the administration of JNK inhibitors to inhibit the JNK/c‐Jun signaling pathway would also inhibit autophagy and apoptosis, but the generation of ROS would not be attenuated by JNK inhibitors. The results indicate that ROS generation is an upstream event of JNK and that autophagy and apoptosis are the key downstream substrates and effectors of the ROS/JNK/c‐Jun signaling pathway. At present, micro‐CT is the best method to evaluate bone microstructure because it is able to clearly show the microstructure of bone trabeculae [21]. We used micro‐CT to evaluate GC‐induced changes in the ONFH model in rats. Compared with those in CG, BV/TV, Tb.N, and Tb.Th decreased in the ONFH group, whereas Tb.Sp increased along with an increase in apoptotic cells and severe bone trabecular lacunae. These results confirm that the ONFH model was successfully established. Compared with the ONFH group, 3‐MAG had better trabecular shape and bone tissue integrity, with only a few empty bone pits and lower apoptosis rate. These findings suggest that the 3‐MA can effectively reduce bone damage and normalize the quantity and shape of bone trabeculae, and this result was consistent with the results of the *in vitro* experiments.

## Conclusions

In summary, GC can induce osteoblast apoptosis and autophagy and contribute to the occurrence and development of ONFH by activating the ROS/JNK/c‐Jun signaling pathway. Furthermore, we provide evidence for the potential therapeutic value of 3‐MA as a treatment for ONFH. Our results might help support new directions for understanding the pathogenesis and developing treatments for ONFH.

## Conflict of interest

The authors declare no conflict of interest.

## Author contributions

PP and HP designed the experiments. PP, ZN, and FS carried out the experiments and analyzed experimental results. PP wrote the manuscript; and HP revised the manuscript. All authors approved the final manuscript.

## Data Availability

The data that support the findings of this study are available from the corresponding author upon reasonable request.
